# Single-Step Detection of the Influenza Virus Hemagglutinin Using Bacterially-Produced Quenchbodies

**DOI:** 10.3390/s19010052

**Published:** 2018-12-23

**Authors:** Hee-Jin Jeong, Jinhua Dong, Hiroshi Ueda

**Affiliations:** 1 Department of Biological and Chemical Engineering, College of Science and Technology, Hongik University, Sejong-si 30016, Korea; heejinjeong@hongik.ac.kr; 2 Key Laboratory of Biological Medicine in Universities of Shandong Province, School of Bioscience and Technology, Weifang Medical University, Weifang 261042, China; dongjh@wfmc.edu.cn; 3 Tokyo Tech World Research Hub Initiative (WRHI), Institute of Innovative Research, Tokyo Institute of Technology, 4259 Nagatsuta-cho, Midori-ku, Yokohama, Kanagawa 226-8503, Japan; 4 Laboratory for Chemistry and Life Science, Institute for Innovative Research, Tokyo Institute of Technology, Yokohama 226-8503, Japan

**Keywords:** influenza A, hemagglutinin, H1N1, Quenchbody, immunosensor

## Abstract

We have successfully generated a Quenchbody that enables the detection of the influenza virus hemagglutinin (HA), in a simple and convenient manner. By two-site labeling of the bacterially-produced anti-HA Fab with ATTO520, its fluorescence intensity was increased to 4.4-fold, in the presence of a nanomolar concentration of H1N1 HA. Our results indicate the potential use of this Quenchbody, as a sensor for the simple in situ detection of influenza A virus.

## 1. Introduction

Influenza, an acute respiratory disease that is induced by the influenza virus, has caused global outbreaks, with severe consequences for human health. Influenza viruses are classified into four subtypes (viz., A through D), on the basis of whether they cause epidemics and induce illness [1]. Clinical attention has been focused mostly on the emergence of subtype A, which causes pandemics. Influenza A virus has genes coding for 1 through 18 hemagglutinin (HA or H) surface proteins (H1–H18; which facilitate a virus attachment to the host cell surface, as well as penetration into cells) and those coding for 1 through 11 neuraminidase (N) surface proteins (N1–N11; which are involved with the release of the viral progeny from the host cells) [2,3]. Interestingly, of the total 198 possible combinations of H and N genes, only three subtypes (viz., H1N1, H2N2, and H3N2, in the order of their emergence) have been found in human-adapted viruses [4]. Among them, the 2009 influenza A H1N1 virus of the swine origin, resulted in the first pandemic of the twenty-first century, with infections leading to an estimated 100,000 deaths, and is currently still circulating worldwide [5]. To control the rapid spread of the influenza A H1N1 virus, along with its globetrotting hosts, there has been a wide interest in the development of accurate detection techniques that will enable a timely diagnosis of the pathogen.

Among the current methods available for the diagnosis of influenza A H1N1 virus, one of the most common tools is the polymerase chain reaction (PCR) with primers or probes that are specifically designed for the H1N1 [6,7,8]. However, the PCR-based method requires laborious steps and complicated procedures, taking a few hours to a day, to obtain results. Thus, PCR-based diagnostics are not quick enough to prevent the spread of the virus, both within and between individuals, and are thus, not ideal in the primary health-care setting. For instance, for patients with a positive test result, any antiviral medicine for the treatment of flu (e.g., Tamiflu) must be taken within two days after the onset of symptoms, in order to begin to eradicate the virus [8]. A recent study used an antibody-based chromatographic approach to qualify the influenza A virus within 30 minutes, indicating this to be an efficient tool for PCR-free detection with the advantage of the experimental procedure being simple and fast [9]. However, this test had a limited sensitivity, and samples from patients with low concentrations of the virus yielded false-negative results. Microfluidic system-based sandwich-type diagnostic assays for the influenza A virus have been performed using mouse monoclonal antibodies, against the virus nucleoprotein, with a detection limit of 0.032 HA units [10]. However, this method also suffers from drawbacks, including its relatively high cost and its sensitivity to temperature and humidity, which results in batch-to-batch variations. Taking these past attempts together, it is obvious that there is still a crucial need to develop a simple and sensitive detection method for the influenza HA.

Previously, we had developed a novel immunosensor protein, named the Quenchbody, which is a site-specific, fluorophore-labeled antibody or its fragment that works on the antigen-dependent removal of fluorescence quenching [11,12,13]. In the absence of an antigen, the fluorescence of the dye is quenched by a photo-induced electron transfer from tryptophan (Trp) residues, in the variable region of the antibody to the dye. After that, when an antigen binds to an antibody, the binding stabilizes the conformation of the antibody variable region and the dye surrounding the antigen-binding site is squeezed out, leading to the dequenching. Therefore, adding a Quenchbody to a target sample and measuring its fluorescence intensity, allow for the antigen to be quantified, without the need for additional reagents or experimental steps. To date, we have applied Quenchbodies as biosensors, for the rapid detection of various targets in a solution, and in some cases on the cells, without performing any washing steps [11,12,13,14,15,16,17,18]. Among these studies, we have developed a Fab-type Quenchbody that recognizes the HAs of the influenza virus H1N1 and H5N1, via a cell-free transcription and translation system [13]. In this case, we labeled the antibody fragments using tRNAs carrying a tetramethylrhodamine (TAMRA)-conjugated phenylalanine (Phe) with an amber anticodon (CUA), and a rhodamine 110-conjugated Phe with four-base anticodon (CCCG). By using this dual-color labeling method, the Förster resonance energy transfer (FRET) between the two dyes induces additional responses to those of dequenching, resulting in a higher signal-to-background ratio than the one obtained through the dequenching of a single-color labeling. However, several issues remained to be solved; namely, the yield of the Quenchbody was limited by the high cost of the cell-free transfection and translation reagent; and the unnatural aminoacyl-tRNAs conjugated with the fluorescent dye, recognized the amber anticodon and the four-base codon. In this present study, we have generated, on a larger scale, a Quenchbody that recognizes influenza HA, by using a combination of the *Escherichia coli* expression and thiol-based, fluorescence-labeling methods. Specifically, we tested a number of fluorescence dyes, as well as the number of their labeling sites, to obtain a high response.

## 2. Materials and Methods

### 2.1. Materials

The KOD-Plus-Neo DNA polymerase and T4 ligase were obtained from Toyobo (Osaka, Japan). The restriction enzymes and the *E. coli* SHuffle T7 Express lysY cells were obtained from New England Biolabs Japan (Tokyo, Japan). The oligonucleotides were obtained from Operon-Eurofins (Tokyo, Japan). The PureYield plasmid miniprep kit was obtained from Promega (Tokyo, Japan). The ultrafiltration devices were obtained from Millipore (centrifugal filter tube Ultra-4, MWCO 3 k; Tokyo, Japan). The immobilized Tris(2-carboxyethyl)-phosphine (TCEP) disulfide-reducing gel was obtained from Pierce Biotechnology (Thermo Fisher Scientific, Rockford, IL, USA). ATTO520-C2-maleimide was obtained from the ATTO-TEC (Siegen, Germany). TAMRA-C5-maleimide was obtained from Biotium (Hayward, CA, USA). The Talon resin was obtained from Clontech (Takara-Bio, Shiga, Japan). The His SpinTrap column was obtained from GE Healthcare (Piscataway, NJ, USA). Anti DYKDDDDK-tag antibody beads and the DYKDDDDK peptide were obtained from Wako Pure Chemicals (Osaka, Japan). The recombinant HA protein from A/California/04/2009 H1N1 was obtained from Sino Biological (Beijing, China). Unless otherwise indicated, all other chemicals and reagents used were from Wako Pure Chemicals or Sigma (Tokyo, Japan).

### 2.2. Gene Constructions

To construct a DNA sequence for generating a single-labeled Quenchbody, we used the pUQ1H vector on which a Cys-tag (MAQIEVNCSNETG) was encoded at the N-terminus of the heavy chain of the antigen-binding fragment (Fab) [13]. To generate the gene for the double-labeled Quenchbody, we used the pUQ2 vector on which two Cys-tags were encoded at the N-terminus of both the heavy and the light chains of the Fab [13]. In parallel, we amplified DNA of either the heavy or light chain of the anti-HA antibody, through PCR, using the synthetic gene-encoding FI6v3 [19] as a template. The primer sets AgeIFI6v3VHback (5′-atgagaccggtggcggttcaggcggcggatcacaggttcagctggtggaatca-3′) and XhoIFI6v3VHfor (5′-aagcgctcgagacggtgactgaggttccttggccccaa-3’), and EcoRvFI6v3VLback (5′-aaggagatatcatatggacattgtgatgactcaga-3′) and HindIIIFI6v3VLfor (5′-ttcaagcttggtgccttggccaaacgtcggtggagt-3′), were used for the PCR amplification of the heavy chain variable region (VH) and the light chain variable region (VL) fragments, respectively, with KOD-Plus-Neo as the enzyme. The PCR-amplified VH and VL sequences were then inserted into *Age*I/*Xho*I- and *Eco*RV/*Hin*dIII-digested pUQ1H vectors, respectively. For insertion into the pUQ2 vector, VL was amplified with the primers SpeIFI6v3VLback (5′-tgagactagtggcggttcaggcggcggatcagacattgtgatgactcaga-3′) and HindIIIFI6v3VLfor, and then cloned into the *Spe*I/*Hin*dIII-digested vector, using the T4 ligase, whereas, VH cloning was done with a protocol similar to that used for pUQ1H.

### 2.3. Fab Expression and Fluorescence-Labeling

*E. coli* SHuffle T7 lysY cells were transformed with each DNA, cultured and induced for protein expression, overnight, at 16 °C, in a 100-mL culture containing 0.4 mM isopropyl-β-d-thiogalactopyranoside, and the cytoplasmic fraction was then recovered. The Fab protein was purified via the His-tag at the C-terminus of its heavy (Fd) chain by an immobilized metal affinity chromatography, using Talon resin. After reduction of the cysteine residue on the Cys-tag(s), using the TCEP agarose beads, labeling with either TAMRA-C5-maleimide or ATTO520-C2-maleimide was performed via the maleimide-thiol reaction. Bearing in mind that, in our previous results, the signal-to-background ratio of the fluorescence response of Quenchbody was highly affected by the removal of the unbound dye [17], we purified the Quenchbody using Ni-NTA resin (His SpinTrap), before the DYKDDDDK-tag affinity purification was performed, as indicated by the manufacturers. The buffer was exchanged to phosphate-buffered saline added with Tween (PBST) (10 mM phosphate, 137 mM NaCl, 3.7 mM KCl, pH 7.2, 0.1% Tween 20) before concentration (by ultracentrifugation), quantitation, and storage of the purified protein.

### 2.4. Fluorescence Measurements

The fluorescence intensity of each Quenchbody (2 nM in 250 µL of PBST) in a quartz microcuvette was measured, using an FP-8500 spectrofluorometer (JASCO, Tokyo, Japan) with various concentrations of H1N1 HA in 2 µL of PBST added for titration. As a control, the same volume of PBST was added to normalize the signal. With excitation at 520 ± 2.5 and 546 ± 2.5 nm for ATTO520- and the TAMRA-labeled Quenchbodies, respectively, fluorescence titration curves were drawn at the emission maxima of each spectrum. The Y axis value of LOD (Y_LOD_) was calculated by using Y_LOD_ = mean_blank_ + 1.645 (SD_blank_) + 1.645 (SD_low concentration sample_) [20] and the corresponding LOD value was determined using GraphPad Prism (GraphPad Software, San Diego, CA, USA).

## 3. Results and Discussion

By using cDNAs for an anti-HA antibody FI6v3, which is a variant of the neutralizing antibody FI6 selected from human plasma B cells that binds to group 1 and group 2 Influenza A HAs [19], we made two types of Fab-based Quenchbody constructs. Since this antibody has more Trp residues (4) in the heavy chain variable region VH than in the light chain V region VL (2), we attempted to make single-labeled Quenchbody by tethering a Cys-tag at the N-terminus of heavy chain in the Fab expression vector. Additionally, a Fab fragment with two Cys-tags, at both chain N-termini, was constructed, expecting a larger quenching and antigen response, due to the dye–dye interaction. These two constructs were used to express the Fab derivatives in *E. coli*, with oxidative cytoplasm, and the soluble proteins were recovered and purified by immobilized metal affinity chromatography. Afterwards, we used two types of fluorescent dyes for the labeling, namely, TAMRA and ATTO520, since these dyes were previously confirmed to be efficiently quenched in anti-osteocalcin Quenchbodies [12,13]. After labeling, the proteins were carefully tandem-purified to remove the excess free dyes, before following the fluorescence measurements.

**Figure 1:**
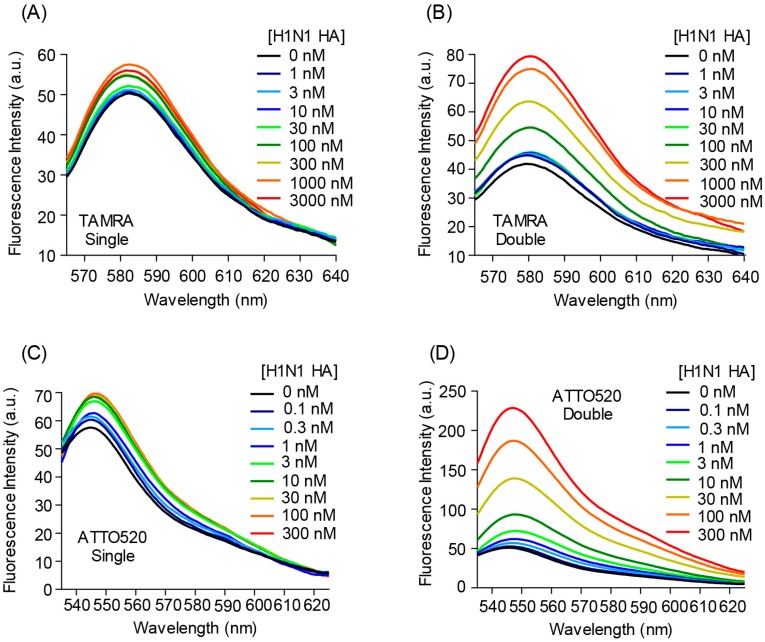
Fluorescence spectra of (**A**) single TAMRA-, (**B**) double TAMRA-, (**C**) single ATTO520-, and (**D**) double ATTO520-labeled anti-hemagglutinin (HA) Quenchbodies, in the presence of H1N1 HA, at the indicated concentrations.

**Figure 2:**
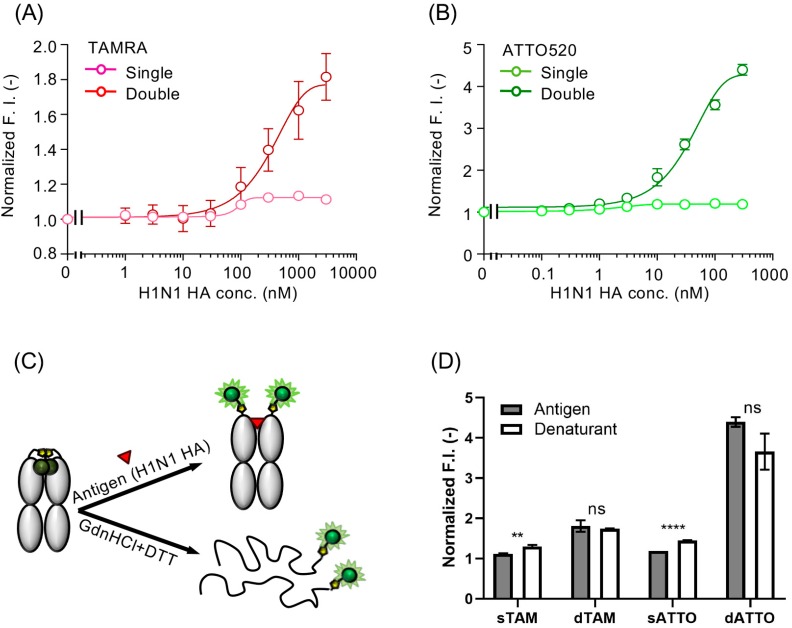
(**A**) Antigen concentration-dependent fluorescent response of the TAMRA-labeled Quenchbody. The normalized fluorescence intensity of each sample, based on the fluorescence intensity at zero-dose, was plotted. Error bars represent ±1 SD (*n* = 3). (**B**) The same as in (**A**), for the ATTO520-labeled Quenchbody. Error bars represent ±1 SD (*n* = 2). (**C**) Schematic representation of a Quenchbody, in the presence of the antigen or denaturants (7 M guanidine hydrochloride (GdnHCl) and 100 mM dithiothreitol (DTT)). (**D**) Normalized fluorescence intensities of the Quenchbodies in the presence of 300 nM H1N1 HA (*n* = 2 or 3) or denaturant (*n* = 3). Statistical comparisons of the data were carried out by Student’s *t*-test using GraphPad Prism software (ns: *p* > 0.05; ** *p* < 0.01; **** *p* < 0.0001).

The fluorescence intensity of the single TAMRA-, double TAMRA-, single ATTO520-, and the double ATTO520-labeled Quenchbodies was increased to 1.12 ± 0.01-, 1.81 ± 0.14-, 1.19 ± 0.00-, and 4.40 ± 0.12-fold, respectively (Figure 1 and Figure 2A,B), in an antigen-concentration-dependent manner. In addition, to evaluate the degree of quenching, these Quenchbodies were denatured using a denaturant (7 M guanidine hydrochloride, 100 mM dithiothreitol, pH 7.0) and the resultant fluorescence intensities were normalized against those obtained under the non-denatured condition (PBST was added as a buffer instead of the denaturant). As a result, the responses of the denatured Quenchbodies were 1.30 ± 0.04-, 1.74 ± 0.01-, 1.45 ± 0.01-, and 3.66 ± 0.45-fold, for the single TAMRA-, double TAMRA-, single ATTO520-, and the double ATTO520-labeled Quenchbodies, respectively, which were similar to those in the presence of the maximum antigen concentration (Figure 2C,D). It is worth noting that the peak emission of the free dyes in the denaturant was 81% and 95% that of those in the PBST, for the ATTO520 and TAMRA, respectively (Supplementary Figure S1). Hence, it is reasonable that the denaturant values of the double labeled Q-body, as shown in Figure 2D, were smaller than the corresponding Antigen values. This result showed that in the case of a double-labeled Quenchbody, the quenched fluorescence was almost fully dequenched by the antigen, indicating that the fluorescence dye was successfully incorporated onto the Fab fragment and the quenching was removed in an antigen-concentration-dependent manner. On the other hand, in the case of a single-labeled Quenchbody, the dequenching efficiency by the antigen was not so high. Interestingly, the maximum responses of the double-labeled Quenchbodies, in the presence of the antigen or the detergent, were much higher than two times those of the single-labeled Quenchbodies. These results suggested that the release of the H-type dimer [21], after dequenching in the presence of the antigen, had affected the signal. That is, the antigen-dependent responses of the single-labeled Quenchbodies started to increase in the presence of a low concentration of antigen, whereas, those of the double-labeled Quenchbodies increased in the presence of a relatively higher concentration of antigen, suggesting that the dequenching of single-labeled Quenchbodies was rapidly switched on, whereas, the “switching” was difficult for the double-labeled Quenchbodies, because of the release of the H-type dimer formation between the two dyes. It is worth noting that these Quenchbodies showed a high sensitivity. That is, the EC_50_ (half-maximal effective concentration) of the single TAMRA-, double TAMRA-, single ATTO520-, and the double ATTO520-labeled Quenchbodies was 0.99 μM, 0.28 μM, 4.9 nM, and 63 nM, respectively. When we focused on the double ATTO520-labeled Quenchbody, which showed the best fluorescence response, its limit of detection (LOD) value [20] was 3.34 ± 0.76 nM, indicating the utility of this Quenchbody as a practical sensor for detecting H1N1 HA, in the nanomolar order sensitivity. According to the manufacturer’s description, the molecular weight of the H1N1 HA is 50 kDa. Thus, the LOD value of the double ATTO520-labeled Quenchbody could be written in a different unit, as 165 ± 38 ng/mL, which shows a similar value to the anti-H5N1 HA Fab (100 ng/mL), for an open-sandwich ELISA [22].

The maximum response values of these Quenchbodies were slightly lower (7-fold) than those of our previous HA Quenchbody for H1N1 [13], which was site-specifically-labeled with amber anticodon-conjugated TAMRA and the four-base, anticodon-conjugated R110, through a cell-free transcription–translation system. The cell-free-based method enables heterolabeling, resulting in a FRET between the dequenched dyes. Moreover, we surmised that the possible reasons for these differences between the previous cell-free-based results and the current *E. coli*-based results are as follows. First, the labeling site, whether on the amber-tag or the Cys-tag, as well as the chemical structure of the linker between the dye and the antibody fragment, was not exactly the same, and thus, the efficiency of quenching and dequenching, with internal Trp residues, showed different values. Second, the disulfide bond between the heavy and light chains was removed to avoid the maleimide-thiol-based labeling, and so it is possible that the Fab had a slightly different conformation. These possibilities are worth further exploration. However, it is worth noting that when we previously compared the activities of the BGP (bone gla protein) Q-bodies made of *E. coli*-expressed and cell-free based Fabs, they showed almost the same response [13], indicating that the *E. coli*-produced Fab fragment devoid of interchain disulfide bond retains its full binding activity, due to the strong noncovalent interaction between the CH1 and the CL domains, even without the disulfide bond between them. We think that it is also the case for this FI6v3 Q-body sharing the same CH1/CL domains.

Although the response of the *E. coli*-based Quenchbody was slightly lower than that of the heterolabeled cell-free-based one, the *E. coli*-based approach for constructing Quenchbodies has several superior advantages, over our previous cell-free-based method. First, the yield of Fab protein produced by *E. coli* is higher than that from the cell-free-based approach, suggesting its possibility to be used for the several low-end applications as an influenza virus sensor. Moreover, the large range of commercial maleimide-conjugated fluorophores is more easily available than the tRNA-conjugated dye used for the cell-free conjugation.

## 4. Conclusions

In this study, we generated Fab-type Quenchbodies that can be used as a sensor for the rapid and handy detection of the influenza virus hemagglutinin (HA). The *E. coli*-based method for generating Quenchbodies enabled improvements of the production yield and cost-efficiency, thereby, showing promise as a reliable means for diagnosing the influenza virus in situ. The notable superiority of this HA Quenchbody approach over conventional immunoassays, for influenza virus, including immunochromatography, is the lack of a need for bound/free separation of the unbound probe for reducing the background signal, resulting in a simple tool for the detection of various influenza viruses.

## Supplementary Materials

The following are available online at http://www.mdpi.com/1424-8220/19/1/52/s1, Figure S1. Peak fluorescence intensity of free ATTO520-COOH (A) and TAMRA-COOH (B) in PBST and denaturant (7 M Gdn-HCl, 100 mM DTT). Excitation wavelength was 520 ± 2.5 nm and 546 ± 2.5 nm, respectively.
