# Supplementary material for: Single-Step Detection of the Influenza Virus Hemagglutinin Using Bacterially-Produced Quenchbodies
Source: Sensors (Basel). 2018 Dec 23;19(1):52. doi: 10.3390/s19010052 (PMC6338965; doi:10.3390/s19010052)
Supplement: Supplementary file 1 [file sensors-19-00052-s001.pdf]

Supplementary Information for

Single-step detection of influenza virus hemagglutinin using bacterially produced Quenchbodies

Hee-Jin Jeong [1], Jinhua Dong [2,3,4] and Hiroshi Ueda [4,*]

[1] Department of Biological and Chemical Engineering, College of Science and Technology, Hongik University, Sejong-si, 30016, Korea; heejinjeong@hongik.ac.kr
[2] Key Laboratory of Biological Medicine in Universities of Shandong Province, School of Bioscience and Technology, Weifang Medical University, Weifang 261042, China; dongjh@wfmc.edu.cn
[3] Tokyo Tech World Research Hub Initiative (WRHI), and
[4] Laboratory for Chemistry and Life Science, Institute for Innovative Research, Tokyo Institute of Technology, Yokohama 226-8503, Japan
* Correspondence: ueda@res.titech.ac.jp; Tel.: +81-45-924-5248

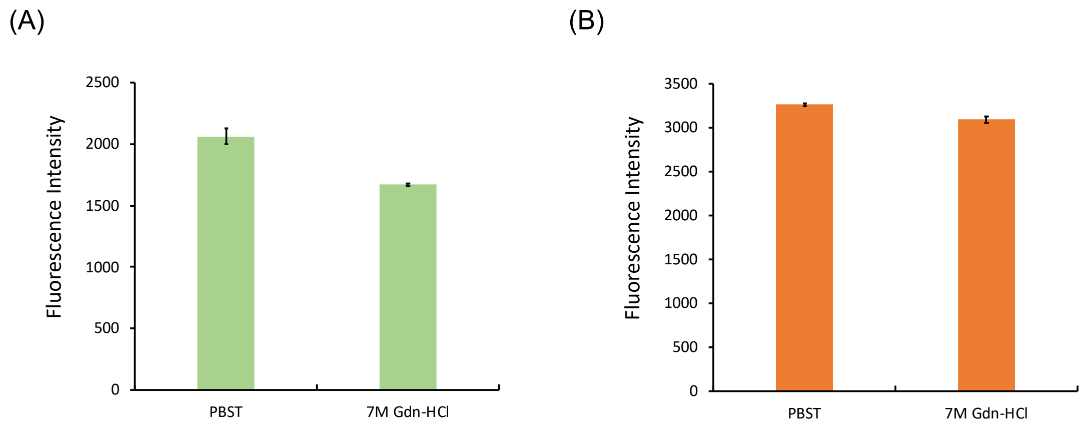

Figure S1. Peak fluorescence intensity of free ATTO520-COOH (A) and TAMRA-COOH (B) in PBST and denaturant (7M Gdn-HCl, 100 mM DTT). Excitation wavelength was 520 ± 2.5 nm and 546 ± 2.5 nm, respectively.
